# Flavoprotein Autofluorescence Imaging of Visual System Activity in Zebra Finches and Mice

**DOI:** 10.1371/journal.pone.0085225

**Published:** 2014-01-06

**Authors:** Neethu Michael, Hans-Joachim Bischof, Siegrid Löwel

**Affiliations:** 1 Systems Neuroscience Group, Bernstein Focus Neurotechnology and Johann-Friedrich-Blumenbach-Institute for Zoology and Anthropology, University of Göttingen, Göttingen, Germany; 2 Neuroethology, University of Bielefeld, Bielefeld, Germany; Universität Bielefeld, Germany

## Abstract

Large-scale brain activity patterns can be visualized by optical imaging of intrinsic signals (OIS) based on activity-dependent changes in the blood oxygenation level. Another method, flavoprotein autofluorescence imaging (AFI), exploits the mitochondrial flavoprotein autofluorescence, which is enhanced during neuronal activity. In birds, topographic mapping of visual space has been shown in the visual wulst, the avian homologue of the mammalian visual cortex by using OIS. We here applied the AFI method to visualize topographic maps in the visual wulst because with OIS, which depends on blood flow changes, blood vessel artifacts often obscure brain activity maps. We then compared both techniques quantitatively in zebra finches and in C57Bl/6J mice using the same setup and stimulation conditions. In addition to experiments with craniotomized animals, we also examined mice with intact skull (in zebra finches, intact skull imaging is not feasible probably due to the skull construction). In craniotomized animals, retinotopic maps were obtained by both methods in both species. Using AFI, artifacts caused by blood vessels were generally reduced, the magnitude of neuronal activity significantly higher and the retinotopic map quality better than that obtained by OIS in both zebra finches and mice. In contrast, our measurements in non-craniotomized mice did not reveal any quantitative differences between the two methods. Our results thus suggest that AFI is the method of choice for investigations of visual processing in zebra finches. In mice, however, if researchers decide to use the advantages of imaging through the intact skull, they will not be able to exploit the higher signals obtainable by the AFI-method.

## Introduction

Optical imaging is a relatively new tool for the investigation of the functional organization of the cortex. The oldest and most commonly used imaging technique is intrinsic signal optical imaging (OIS), which is dependent on the oxygenation and deoxygenation of hemoglobin [1]. A more recently developed technique is the so-called flavoprotein autofluorescence imaging (AFI), which is based on the two electron carriers flavin adenine dinucleotide and flavin mono nucleotide, both associated with the mitochondrial electron transport chain [2]. While both techniques have a temporal resolution too low to study the dynamics of cortical processing [3], they are ideally suited to monitor the activation of whole brain areas in a rather non-invasive way. Measurements of neuronal activity in the visual system using imaging of intrinsic signals was initially developed by Grinvald et al. [4] and recently modified by Kalatsky and Stryker [5]. OIS is based on the measurement of oxygenation changes of the local blood supply of the nervous tissue. It is assumed that oxygenation is inversely proportional to the ongoing metabolic activity of the neurons. The activation of neurons enhances their metabolic activity and thus the consumption of oxygen. This in turn leads to an increase of deoxyhemoglobin in adjacent blood vessels followed by an enhanced influx of fresh oxygenated blood. The changes of the hemoglobin oxygen content lead to changes of the scattering of incident light and thus to alterations in the intensity of the light reflected from the tissue. This “intrinsic signal” is captured by a sensitive CCD camera, and amplified and analyzed by a computer program.

Flavoprotein autofluorescence imaging is based on the fluorescence of oxygenated mitochondrial flavoprotein. Neural activity leads to an enhancement of intracellular Ca^++^ and the aerobic energy metabolism. This enhancement triggers the transformation of mitochondrial flavoproteins from a reduced non-fluorescing form into an oxidized form [2], [6], [7], which shows green fluorescence with a peak emission at ∼520 nm when excited with blue light [8]. It has been found that there is a linear relationship between the fluorescence signals and neuronal activity, and this makes flavoprotein fluorescence imaging a highly suitable tool for visualizing neuronal activity [2], [9]. It has also been shown that there is no or reduced vascular artifacts and hence it can be considered advantageous when compared to OIS based on the local blood flow [9].

The zebra finch is a frequently used model for visual system investigations in laterally eyed birds [10], [11]. Its visual system, like that of all vertebrates, comprises two main visual pathways originating from the retinal ganglion cells [12] which are called the tectofugal and the thalamofugal projection in birds. The avian tectofugal projection is homologue to the so-called extrageniculate pathway in mammals [12], [13]. The second projection is called thalamofugal pathway, leading from the retina to a thalamic cluster of cells and then to the visual part of the hyperpallium, a layered structure at the dorsal pole of the telencephalon. This structure is also called “visual wulst”, and is discussed as a homologue of the mammalian visual cortex. Accordingly, the thalamofugal pathway is hypothesized to be homologous to the geniculocortical pathway of mammals [12]–[14].

We have recently used OIS for the visualization of neuronal activity in the visual wulst of zebra finches [15]. Our experiments have demonstrated that the visual wulst comprises one or even more retinotopic maps representing the visual field of the contralateral eye. The avian wulst is thus not only very similar to the mammalian visual cortex based on its circuitry and physiological properties [13], but may also be similar in its architecture and function [16]. Although the similarities are quite striking, it is still under discussion how far the similarities between the avian wulst and the mammalian cortex justify a denomination as homologue [14]. We therefore started a detailed study of the fine organization of the zebra finch visual wulst, to compare it with the existing data from the mouse visual cortex.

Because vascular artifacts were often a problem in our previous study using OIS, we established AFI, which has not yet been used in bird studies. Indeed, this technique led to nearly vascular artifact-free cortical maps, and the overall signal strength and quality of the retinotopic maps obtained by AFI was significantly higher compared to maps recorded by OIS. To verify this impression, we conducted a quantitative study comparing the two methods. We also included mouse experiments into this study because the main aim of our ongoing studies is a comparison of the function of visual areas in birds and mammals. The advantage of the flavoprotein method over the intrinsic signal imaging was supported by our experiments in zebra finches. But, to our surprise, no significant difference between the two methods could be obtained in the case of mouse visual cortex imaging which we routinely performed through the intact skull. We then presumed that methodological differences between the mouse and the zebra finch experiments led to this difference. Indeed, when we craniotomized mice before the experiment the flavoprotein imaging technique also provided higher signal amplitudes upon sensory stimulation in the visual cortex of mice.

## Materials and Methods

Eight zebra finches of 100–110 days of age from the breeding stock of Bielefeld University and sixteen adult C57Bl/6J mice of both sexes aged 110–115 days obtained from the mouse colony of the central animal facility of University Medical Center, Göttingen were used for the present study.

### Ethics Statement

All experimental procedures were performed according to the German Law on the Protection of Animals and permitted by the local government: Niedersächsisches Landesamt für Verbraucherschutz und Lebensmittelsicherheit and approved by the Tierschutz Kommission des Landes Niedersachsen nach § 15 TSG. (Permission no 84-02.04.2011.A217).

### Surgical Preparations for Optical Imaging

The birds were anesthetized by injecting 0.1 ml of 20% urethane intramuscularly. The birds were then kept under infrared light to maintain the body temperature. Using the stereotaxic head holder for small birds [17], the birds were fixed. The head feathers were then removed and the skin covering the skull was locally anesthetized with 2% xylocaine jelly. After 2 min, the skin was carefully cut off to expose the skull. A craniotomy was then performed on the left hemisphere to expose the visual wulst, leaving the dura mater intact. Care was always taken to keep the brain surface moist. Warm agarose (2.5% in saline) and a glass cover slip were used to cover the exposed brain area. The agarose borders were then decorated with petroleum jelly to prevent drying of the agarose. The contralateral eye was opened by retracting and removing the lower eyelid, keeping the nictitating membrane intact. The nictitating membrane is functioning even in deeply anaesthetized birds and prevents the sclera from drying. If moistening by the nictitating membrane appeared to be not sufficient, silicon oil was applied to keep the sclera in good condition.

The mice were initially box-anesthetized with 2% halothane in a mixture of O_2_:N_2_O (1∶1) and received an injection of atropine (Franz Köhler, 0.3 mg/mouse, subcutaneously), dexamethasone (Ratiopharm, 0.2 mg/mouse, subcutaneously), and chlorprothixene (Sigma, 0.2 mg/mouse, intramuscularly). In addition, lidocaine (2% xylocain jelly) was applied locally to all incisions. The animals were placed in a stereotaxic frame. The animal’s body temperature was maintained at 37°C and continuously monitored using a rectal probe and heart rate was monitored throughout the experiment. Inhalation anesthesia was maintained with 0.6%–0.8% halothane in a mixture of O_2_:N_2_O (1∶1). The skin above the skull was incised to expose the primary visual cortex (V1). In craniotomy experiments, the skull overlying V1 was then removed. In all cases, the dura mater was kept intact. Care was taken to keep the skull and brain surface moist. The exposed area was covered by agarose and a glass coverslip. The agarose borders were then coated to prevent it from drying and to avoid illumination of the brain from the side. The eyes were always kept moist by applying silicon oil.

### Optical Imaging

**Figure 1:**
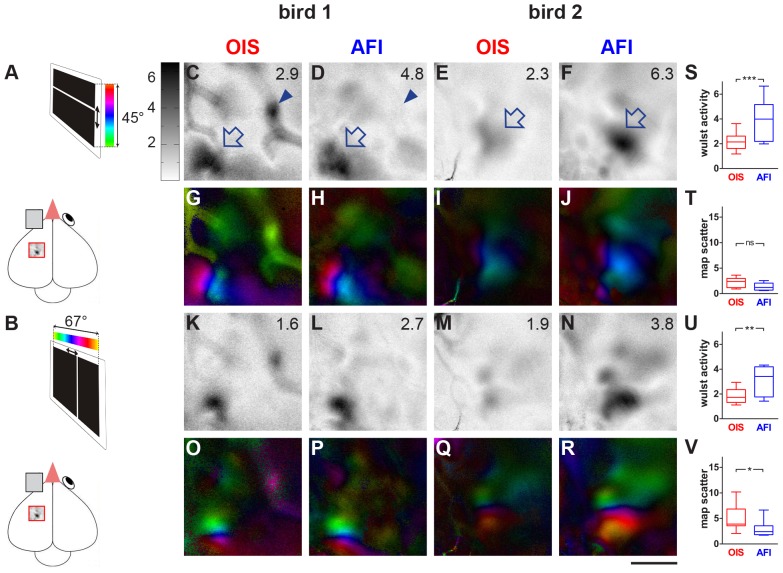
AFI-recordings yield higher magnitude activity maps in zebra finch compared to OIS. Gray-scale coded response magnitude maps (C–F, K–N) and color-coded polar maps of retinotopy (G–J, O–R) of two different birds are illustrated. Wulst activation is displayed as fractional change in reflection ×10^−4^: Darker grey values indicate higher wulst activation. For the illustrated maps, the magnitude of activation is quantified as a number in the upper right corner. In addition, the quantification of all recorded maps in all birds (n = 8) is displayed (S–V). Zebra finches were either visually stimulated with a moving horizontal (elevation maps, C–J) or vertical bar (azimuth maps, K–R) and activity and retinotopic maps were recorded by both AFI and OIS in the same individuals. **A,B:** Schematic diagram of the visual stimulation condition showing the zebra finch brain and the stimulus monitor. Activity in the visual wulst of the left hemisphere was recorded after stimulating the right eye with the elevation and azimuth stimulus, while the left eye remained closed. **C–F:** The dark patches indicated by the open arrows correspond to regions with increased neuronal activity induced by the elevation visual stimulation with moving horizontal bars, obtained by either OIS (**C,E**) or AFI (**D,F**). The small dark patch in C, labelled with the arrowhead, corresponds to a vascular artifact, invisible in the AFI-recording in D. **G–J:** Retinotopic elevation maps obtained via OIS and AFI. **K–N:** Azimuth activity maps recorded by OIS and AFI. **O–R:** Retinotopic azimuth maps obtained via OIS and AFI. **S–V:** Quantification of wulst activation (S, U) and retinotopic map quality (T, V,). Scale bar ** = **1 mm.

**Figure 2:**
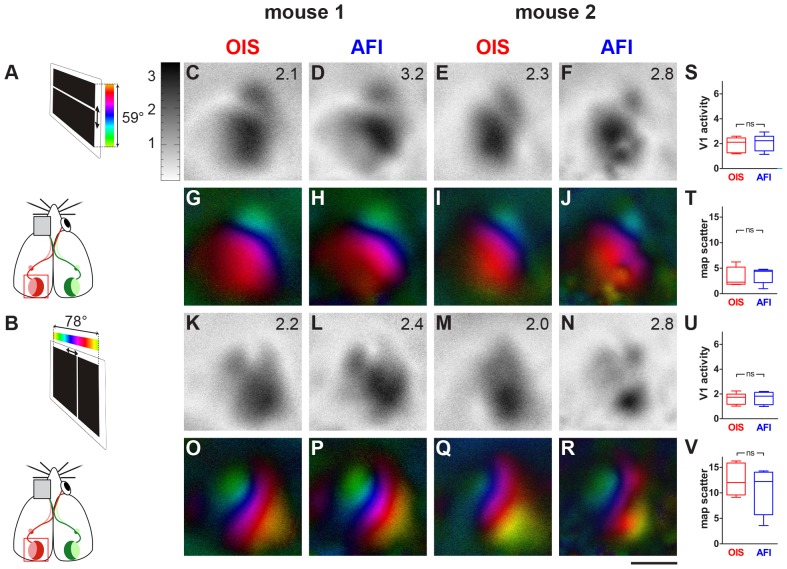
AFI- and OIS-recordings from the visual cortex of mice with intact skull. Data displayed as in Figure 1. Schematic diagram of the visual stimulation condition showing the mouse and the stimulus monitor (A,B). Activity and retinotopic maps recorded with either AFI or OIS appeared very similar (C–R). Similarly, quantitative analyses of the maps did not reveal any significant differences between the two imaging techniques (S–V): both V1-activation and retinotopic map quality were rather similar. Scale bar ** = **1 mm.

Neuronal activity in the visual wulst of zebra finches and in the visual cortex of mice was recorded using both OIS and AFI in the same individuals [2], [9]. For data acquisition and analyses we used the imaging variant introduced by Kalatsky and Stryker [5]. Briefly, for imaging sensory-driven activity a temporally periodic stimulus was continuously presented to the animal, and the response at the stimulus frequency was extracted by Fourier analysis. Optical images of visual cortical or wulst activation were obtained using a CCD camera (Dalsa 1M30) and a 130×55 mm lens with an aperture of 1.2 (Nikon, Tokyo, Japan), controlled by custom software. The acquired image covered 4.5 mm^2^ of the brain surface in both mice and zebra finches. A high refresh rate monitor was placed laterally in a position that the fovea was directed to the center of the screen, at an angle of 60° to the eye contralateral to the imaged hemisphere in the bird experiments (Flatron LCD 295LM 100 Hz, 46.5×30 cm corresponding to 67°×45° visual field; fig. 1A–B), whereas in the mouse experiments the screen was placed at 45° to the eye contralateral to the imaged hemisphere (Hitachi, ACCUVUE, HM-4921-D; 40.6 x30.5 cm ≙ 78×59°; fig. 2A–B). The distance of the screen from the eyes was always maintained at 35 cm in case of zebra finches and at 25 cm in mouse experiments. An azimuth stimulus (vertical bar, 4° wide, moving from 0°–180°) and an elevation stimulus (horizontal bar, 4° wide, moving from 90°–270°) were used for visual stimulation, presented at a temporal frequency of 0.125 Hz. The surface vascular pattern was visualized with illumination wavelengths set by a green (550±10 nm) interference filter using a cold light source (Zeiss KL 2500). After acquisition of the surface image, the camera was focused 600 µm below the cortical surface in mice and in a depth of 500 µm in zebra finches, and neuronal activity was captured using red light (610±10 nm) in case of OIS and blue light (455±10 nm) for AFI. Frames were acquired at a rate of 30 Hz, binned to 7.5 Hz and stored as 512×512 pixel images after spatial binning of the camera image. An additional red respectively blue filter was interposed between the brain and the CCD camera.

### Data Analysis

Two parameters were quantified for the present study: the magnitude of the optical signal as a measure for the magnitude of neuronal activation, and the map scatter which describes the smoothness or regularity of the retinotopic maps. Both parameters were measured separately for azimuth and elevation maps in both zebra finches and mice. Activity maps were calculated from the acquired frames by Fourier analysis to extract the signal at the stimulation frequency using custom software [5]. While the phase component of the signal was used for the calculation of retinotopy, the amplitude component represents the intensity of neuronal activation, i.e. response magnitude expressed as fractional change in reflectance ×10^−4^ [18]. Retinotopic maps were color-coded so that neuronal activation within the brain area observed could be correlated with the position of the stimulus on the monitor eliciting this activation. The combined information of the magnitude of neuronal activation and retinotopy is displayed in so-called polar maps. To evaluate the quality of the retinotopic maps, we used the calculation introduced by Cang et al. [19]. Both the elevation and azimuth maps were used to select the most responsive pixels in the region of interest. For each of these pixels, the difference between its position and the mean position of its surrounding 25 pixels was calculated. For maps of high quality the position difference is small due to smooth progression. The standard deviation of the position difference which is known as map scatter is used as an index of the quality of the retinotopic maps. Lower values indicate lower map scatter and thus higher map quality and higher values indicate lower map quality.

### Statistics

The sample size was 8 in both zebra finch and each of the mouse experiments. One-way repeated measures ANOVA followed by Newman-Keuls multiple comparison tests was applied to compare the map quality and magnitude of activity obtained using either OIS or AFI. The levels of significance were set as “*”, *p*<0.05; “**”, *p*<0.01; and “***”, *p*<0.001.

## Results

At the beginning of our experiments we tried optical imaging of intrinsic signals without craniotomy in zebra finches because the technique is much easier and yields excellent results in mice [20]–[22]. However, it was not possible to visualize any brain activity pattern through the skull, probably due to the special construction of the avian skull (see discussion).

Figure 1 shows examples of activity and retinotopic maps from the visual wulst of two zebra finches recorded by both OIS and AFI after craniotomy. Optical recordings were made in the hemisphere contralateral to the stimulated eye/visual field (fig. 1A,B). The small red rectangle in the scheme of the zebra finch brain shows the approximate position of the recorded visual wulst area. Both elevation maps (figs. 1C–J) elicited by visual stimulation of the bird with a horizontal stripe moving upwards and downwards on the monitor (fig. 1A) as well as azimuth maps (figs. 1K–R), elicited by a vertical stripe moving horizontally on the stimulus monitor (fig. 1B) are illustrated. Comparison of the maps clearly shows differences in signal strength and signal-to-noise-ratio between the two recording techniques: For both elevation and azimuth maps, maps recorded by AFI showed fewer blood vessel artifacts and a significantly higher activation of the visual wulst. For example, in the elevation map of figure 1C, recorded by OIS, blood vessel artifacts strongly contaminate the brain activity pattern. There are two dark regions in the lower left and the upper right of the illustrated wulst region. While the lower left patch is most likely due to a sensory-driven activation of neurons, the upper right patch is most probably a vascular artifact because it is located on a blood vessel. In fact, this dark region is absent in the elevation map recorded by AFI (fig. 1D). Quantification of the imaging data revealed that the magnitude of the wulst activation, which is indicated as a number in the upper right corner of each activity map, was also significantly higher using AFI- compared to OIS-recordings. Figures 1E and F show another example with a visible difference in the magnitude of neuronal activation obtained using OIS and AFI, respectively: in the map recorded by AFI, the activity patch is much darker (fig. 1F), corresponding to a higher wulst activation compared to the OIS-map of the same wulst region (fig. 1E).

Figures 1G to J illustrate so-called polar maps of retinotopy in which the color encodes the position in the visual field and the brightness encodes the magnitude of the visual response. The lower left activity patch shown in figures 1G and H is corresponding to a well-organized retinotopic map, and the vascular artifacts are visible in the OIS-recording (1G) in green; in contrast, in the AFI-recording (1H), these artifacts are much reduced while the retinotopic map is also clearly visible. Figures 1I and J depict another example of polar maps, in which the retinotopic map is not well demarcated in the OIS- recording (1I), but much clearer in the AFI recording (1J).

The azimuth maps recorded from the same birds (figures 1K to R) confirmed our conclusions drawn from the elevation map recordings: again, visual wulst activation was higher in AFI- compared to OIS-recordings (compare figures 1K and L and 1M and N), and vascular artifacts were reduced. The comparison of the polar maps in figures 1O–R obtained by OIS or AFI again showed that the retinotopic maps in AFI-recordings were more clearly demarcated and less noisy compared to the OIS-recordings.

Quantitative analysis of the zebra finch imaging data showed that activity values were nearly doubled in AFI- compared to OIS-recordings (figs. 1S–V). The average wulst activation after visual stimulation with moving horizontal bars (elevation maps) was 2.2 for OIS and 3.9 for AFI-recordings. Similarly, wulst activation elicited by visual stimulation with vertical bars (azimuth maps) was 1.9 for OIS and 3.1 for AFI-recordings. Sensory-evoked wulst activation was significantly different between the two techniques (ANOVA, F (3, 7)  = 17.00, p>0.0001) for both elevation (fig. 1S) and azimuth maps (fig. 1U). The Newman Keuls test showed that the average activation of elevation maps was significantly higher (p = 0.001) using the AFI-technique (mean 3.9, SEM 0.55) compared with that of OIS-experiments (mean 2.2, SEM 0.25). The same was true for the azimuth maps (fig. 1U, OIS: mean 1.9, SEM 0.2; AFI: mean 3.1, SEM 0.39; p<0.01).

The average map scatter value of elevation maps was 2.2 (SEM 0.3) for OIS and 1.4 (SEM 0.2) in case of AFI. However, this difference was not significant (fig. 1T). In contrast, the azimuth map scatter recorded with AFI was significantly smaller (fig. 1V, mean 3.0, SEM 0.5; p<0.05) than the one recorded by OIS (mean 5.0, SEM 0.9). Comparing the quality of the retinotopic maps also showed a significant difference between groups (ANOVA, F (3, 7)  = 6.594, p = 0.0026). It should be noted, however, that the two measures are not independent of each other, i.e. that map scatter is inversely proportional to the magnitude of the cortical responses. The quantitative analyses thus confirmed the impression obtained by visual inspection of the images, namely that in the zebra finch, visual wulst activity was higher and map scatter lower (i.e. retinotopic maps were clearer) in optical recordings with the AFI- compared to the OIS-method.

Figure 2 shows examples of activity and retinotopic maps from the visual cortex of two C57Bl/6J mice recorded by both OIS and AFI through the intact skull. To our surprise and unlike the zebra finch recordings, we were not able to find significant differences between OIS- and AFI-maps, neither in the magnitude of the recorded signals nor in the map quality. This was already quite obvious by pure visual inspection of both the activity maps (compare figs. 2C–D, 2E–F, 2K–L, and 2M–N) and retinotopic polar maps (compare figs.2G–H, 2I–J, 2O–P and 2Q–R). There was no obvious difference between the two imaging techniques in the activity levels, no difference in the appearance of vascular artifacts and in the clarity of the topographic map borders, and also the magnitude of the neural activity did not differ substantially in this example.

The impression obtained by visual inspection of the imaged maps was confirmed by the quantitative evaluation of the recorded data. Although ANOVA indicated a difference between groups (OIS, AFI) in the experiments in mice with intact skull (F (3, 7)  = 8.982, p = 0.0005), there was no significant difference (Newman-Keuls, p>0.05) either in the elevation maps or in the azimuth maps. This was true for both the magnitude of neuronal activation (elevation maps, fig. 2S; OIS: mean 1.9, SEM 0.2; AFI: mean 2.1, SEM 0.2; azimuth maps, fig. 2U, OIS: mean 1.6, SEM 0.2; AFI: mean 1.7, SEM 0.2) and the map scatter (fig. 2T, elevation map scatter, OIS: mean 6.6, SEM 3.1, AFI: mean 5.0, SEM 1.4; fig. 2V, azimuth map scatter, OIS: mean 14.0, SEM 1.7, AFI: mean 10.5, SEM 1.6).

**Figure 3:**
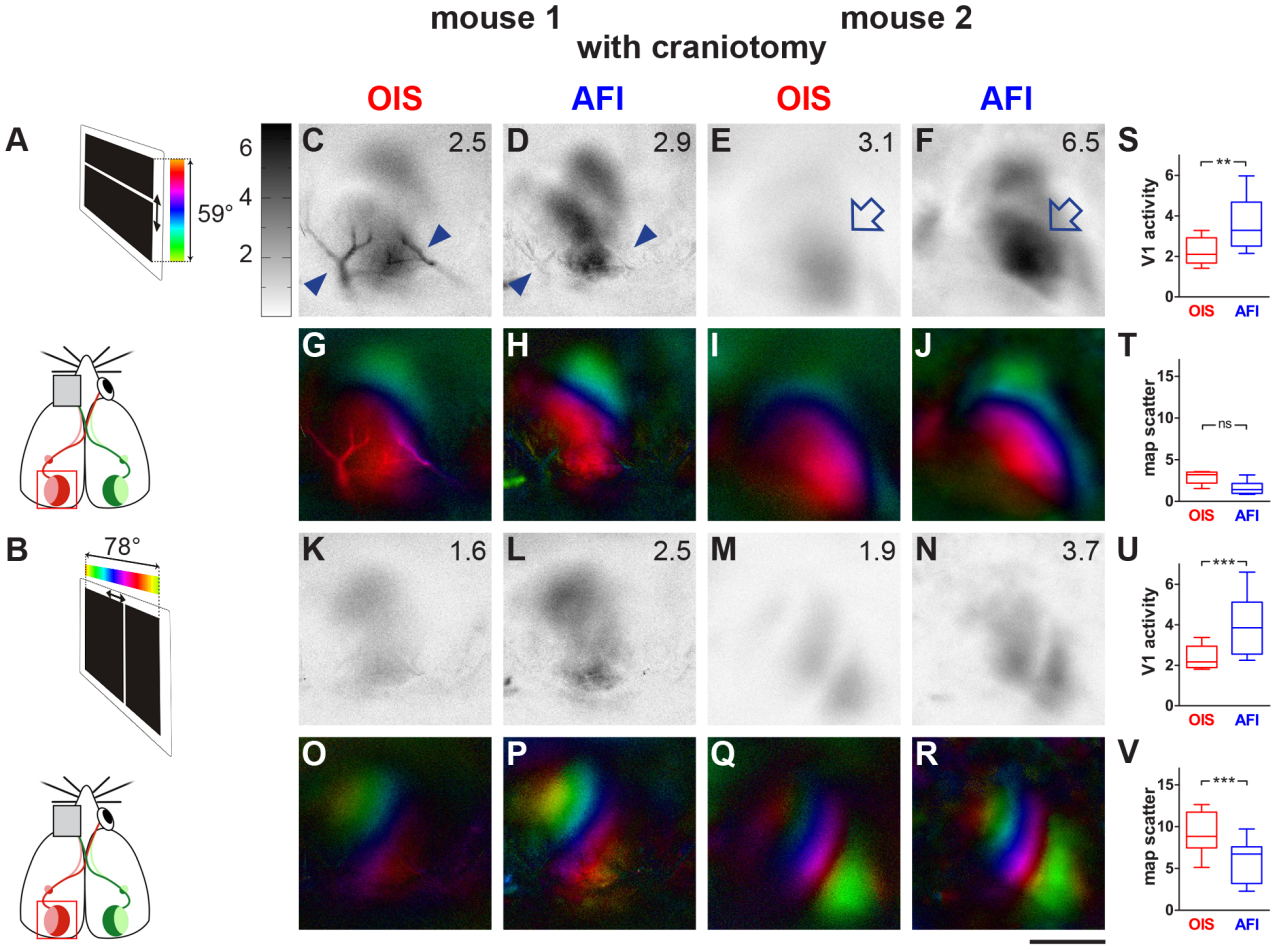
AFI-recordings yield higher magnitude activity maps after craniotomy in V1 of mice compared to OIS. Data displayed as in Figure 1. Schematic diagram of the visual stimulation condition showing the mouse and the stimulus monitor (A,B). As observed in the visual wulst of zebra finches, activity maps (open arrows in E,F) recorded with AFI had higher amplitude and retinotopic maps had lower map scatter compared to OIS. In addition, blood vessel artifacts (labelled by the arrowheads) were reduced in AFI-recordings (compare Figs. 3C and D). Scale bar ** = **1 mm.

While optical recordings through an intact skull in mice did not reveal any differences between OIS and AFI, differences reminiscent of the zebra finch results were obtained after craniotomy in mice. Examples of activity and retinotopic maps from the visual cortex of two other C57Bl/6J mice recorded by both OIS and AFI after craniotomy are illustrated in Figure 3. In the OIS-recorded elevation map (fig. 3C), vascular artifacts were quite strong while they were almost absent in the AFI-recorded activity map (fig. 3D). There was also an obvious difference in the magnitude of the recorded signals between AFI and OIS: V1-activation was much higher in AFI-maps, visible in the darker activity patches compared to the OIS-maps. The average V1-activation is indicated as a number in the upper right corner of each activity map (figs. 3C–D and 3E–F). The retinotopic maps (figs. 3G, OIS and 3H, AFI) also showed a reduction of vascular artifacts and a better map quality, i.e. reduced scatter in the maps obtained by AFI (figs. 3I and J) compared to OIS. The azimuth maps confirmed these results. In the illustrated examples, vascular artifacts were less obvious and thus a reduction by AFI was not observed. However, the magnitude of activity in the recorded maps was clearly higher with AFI (fig. 3L and N) compared to OIS (fig.3K and M), and the retinotopic maps were much clearer and better differentiated in the AFI- (fig. 3P and R) compared to the OIS-recordings (fig. 3O and Q).

Quantitative analysis of the mouse imaging experiments after craniotomy confirmed a significant difference in both neuronal activity and map quality between the two techniques. The magnitude of neuronal activation in V1 upon sensory stimulation was significantly different between groups (ANOVA, F (3, 7)  = 12.26, p = 0.0001). V1-activation after visual stimulation with moving horizontal bars (elevation maps) was significantly higher in AFI- (mean 3.6, SEM 0.4; p<0.01, Newman-Keuls) compared to OIS-recordings (mean 2.2, SEM 0.2; fig. 3S). Similarly, V1-activation after visual stimulation with moving vertical bars (azimuth maps) was significantly higher in AFI- (mean 3.9, SEM 0.5; p<0.001) compared to OIS-recordings (mean 2.4, SEM 0.2; fig. 3U). Results similar to our zebra finch experiments were obtained when the elevation map scatter was compared (fig. 3T): V1-map quality in mice tended to be better (the map scatter was lower) in AFI-recordings (mean 1.6, SEM 0.3) compared to OIS (mean 2.9, SEM 0.3), but the difference was statistically not significant (p>0.05). In contrast, azimuth map quality was significantly better in case of AFI (mean 6.1, SEM 0.9; p<0.001) compared to OIS (mean 9.3, SEM 0.8; fig. 3V).

## Discussion

Previous investigations from our laboratory have already shown that retinotopic representations of visual space can be demonstrated by OIS within the visual wulst of the zebra finch, a small songbird with laterally placed eyes [15]. Here we show for the first time that flavoprotein autofluorescence imaging (AFI) can also be used to visualize brain activation in superficial areas of the avian brain. Moreover, AFI-imaging is superior to the OIS-method because it provides higher activation values, lower map scatter, clearer images and reduced vascular artifacts, as already described by Tohmi et al. [9] for recordings in mice. We also report here that transcranial recordings are not possible in birds, and quantitatively demonstrate that in mice the advantage of the AFI-method can only be exploited in craniotomized animals.

There are several speculations why the AFI-signal has such advantages over the OIS-signal. Experiments by Shibuki et al. [2] support the hypothesis that the autofluorescence responses are more directly triggered by neural activity and the resulting increase in Ca^++^ which raises the aerobic energy metabolism. The calcium increase enhances the oxidation of flavoproteins of the neuronal mitochondria which then emit green fluorescent light. The argument that it could also be a glial response which leads to higher activation with the AFI-method was refuted by studies on the role of flavoproteins in the lactate production which is different in neurons and glia [23], [24]. AFI is thus bound to the region of interest since it is confined to the activated neuron. Oxygenation changes in the blood vessels are therefore not detected (although occurring only milliseconds later), and this results in the notable mitigation of vascular artifacts [25]. When we compared AFI and OIS for visualizing retinotopic representations in the mouse visual cortex, we initially did not find a significant difference in the quality of the obtained images. We then hypothesized that this result could be due to a technical difference in the optical recordings performed in mouse and zebra finch, namely the fact that we performed all bird recordings with craniotomized animals while the mouse recordings were routinely performed through the intact skull. We therefore also started experiments with craniotomized mice, and then found indeed significant differences between AFI and OIS, similar to those obtained in the bird studies.

The idea that AFI is only better than OIS in craniotomized preparations is also supported by the study of Husson et al. [26] who examined the usefulness of AFI for investigations of visual function in mice and cats. In this study, it was shown that the spatiotemporal profile of the AFI-signal had advantages over OIS-imaging, including spatially restricted fluorescence throughout its response duration, reduced susceptibility to vascular artifacts, an improved spatial response profile and a faster time course. According to this study, there is also a twofold increase in the recorded neuronal activity. However, these claims were mostly based on experiments with craniotomized cats while in the mouse experiments; an advantage of the AFI-method was not obvious. Moreover a quantitative demonstration of the results was not given.

OIS in mice with intact skull has become the standard technique because it has the advantages of easier preparation, less invasiveness and the potential of repetitive measurements in the same animal [22], [27]. We initially tried to also install this technique for zebra finches, but realized that it was not possible to detect any activity patterns at the brain surface with an intact skull. This is most probably due to the special lightweight “sandwich” construction of the skull in zebra finches as an adaptation to flight. The skull is composed of two thin parallel bone layers building a pneumatized space with crisscrossing tiny bone bridges for structural strength. In our optical imaging attempts without craniotomy, it was only the tiny bone bridges which could be seen in any wavelength and which were obscuring any view to the surface of the brain. Thus, for birds, craniotomy may be the only way to use optical imaging, either using intrinsic signals or flavoprotein fluorescence.

For mice, our study shows that the OIS-technique does not yield better results after craniotomy, but the AFI-technique obviously improves the results in craniotomized animals. Most likely the skull is acting like an additional filter which is effective in AFI-experiments but not with OIS. The reason may be the difference in excitation wavelength that is used for the two methods. While AFI uses blue light (400 nm), the OIS-technique works with the longer wavelength red light (610 nm). It is well known that longer wavelengths are penetrating tissue better than shorter ones and are therefore more frequently used in modern imaging techniques like near infrared spectroscopy. The advantage of longer wavelengths to penetrate the bone of the skull has been shown quantitatively by Hartwig and van Veen [28] for different vertebrate species. Researchers using optical imaging techniques have thus to decide whether they want to use the advantages of AFI at the expense of the extra effort and the disadvantages of craniotomy. For a lot of experimental goals, OIS is obviously the technique of choice; besides its dependence on craniotomy, AFI has been shown to have other demerits. It is suitable for small brains, rather than larger brains like those of primates [2], and it is vulnerable to motion artifacts produced by larger animals through breathing or heart beats [29]. But for birds, as our study shows, AFI is the method of choice.
